# Risk factors associated with comorbid asthma in patients with chronic rhinosinusitis with nasal polyps: a cross-sectional study

**DOI:** 10.1186/s12890-022-02138-0

**Published:** 2022-09-07

**Authors:** Fangyuan Li, Xuechen Wang, Shen Shen, Kai Huang, Ming Wang, Xiaofang Liu, Chengshuo Wang, Jianmin Jin, Luo Zhang

**Affiliations:** 1 Department of Respiratory and Critical Care Medicine, Beijing Tongren Hospital, Capital Medical University, No. 1, DongJiaoMinXiang, DongCheng District, Beijing, 100730 China; 2 Department of Otolaryngology Head and Neck Surgery, Beijing Tongren Hospital, Capital Medical University, No. 1, DongJiaoMinXiang, DongCheng District, Beijing, 100730 China; 3 Beijing Key Laboratory of Nasal Diseases, Beijing Institute of Otolaryngology, Beijing, 100005 China; 4 Department of Allergy, Beijing Tongren Hospital, Capital Medical University, Beijing, 100730 China; 5 Research Unit of Diagnosis and Treatment of Chronic Nasal Diseases, Chinese Academy of Medical Sciences, Beijing, China

**Keywords:** Chronic rhinosinusitis, Nasal polyps, Comorbid-asthma, Risk factors, Asthma screening system

## Abstract

**Background:**

Although 20–60% of patients with chronic rhinosinusitis with nasal polyps (CRSwNP) have asthma, the risk factors associated with comorbid asthma are not clear. The aim of the study was to investigate the factors associated with asthma, and develop a practical scoring system to screen asthma comorbidity in CRSwNP patients.

**Methods:**

This report describes a cross-sectional study with consecutive CRSwNP patients. Two cohorts of CRSwNP patients named “modelling” group and “validation” group were investigated respectively. Logistic regression analysis was performed based on demographic and clinical data collected from patients in the modelling group to determine the risk factors associated with asthma, and establish a scoring system for screening comorbid asthma. Receiver operating characteristic curve was constructed to evaluate the screening system; the optimal cut-off point was established by means of the Yoden Index. The consistency between the diagnosis of asthma by the Global Initiative for Asthma (GINA) criteria and by the screening system was assessed by Kappa value in the validation group.

**Results:**

Totally 150 patients in modelling group and 78 patients in validation group were enrolled. Female gender (odds ratio [OR] = 6.4; *P* < 0.001), allergic rhinitis (OR = 2.9; *P* = 0.021), serum total (T)-immunoglobulin (Ig) E ≥ 69.0kU/L (OR = 12.0; *P* < 0.001), and blood eosinophil count  ≥ 0.35 × 10^9^/L (OR = 4.0; *P* = 0.001) were shown to be independent risk factors for asthma in patients with CRSwNP. Based on these variables, a scoring system (FAIE) ranging from 0(no risk) to 6(high risk); was developed. The area under the receiver operating characteristic curve of the system was 0.823, and the optimal cut-off value was 3 points, with sensitivity 83.8% and specificity 68.6% for screening asthma. The asthma comorbidity determined with FAIE score ≥ 3 points in the validation group, was moderately consistent with that defined by GINA (Kappa = 0.513, *P* < 0.001), with sensitivity 76.9% and specificity 74.4%.

**Conclusions:**

Female gender, allergic rhinitis, serum T-IgE level, and blood eosinophil count are independent risk factors for asthma comorbidity in patients with CRSwNP, and the FAIE system may be practical for screening comorbid asthma in these patients.

## Background

Chronic rhinosinusitis with nasal polyps (CRSwNP) is a chronic inflammatory disease of the nose and paranasal sinuses, which is present in 2–4% of the adult population [1–3]. About 20–60% of patients with CRSwNP also have comorbid asthma [3–5]; with the typical clinical picture of patients with CRSwNP and comorbid asthma characterized by older age, higher incidence of allergic rhinitis, longer duration of nasal symptoms, higher computed tomography (CT) and endoscopy scores, higher number of sinonasal surgeries, and bronchial obstruction [6–8]. Indeed, in patients with non-atopic asthma and late-onset asthma, CRSwNP was found even more frequently [9]. Moreover, Bilodeau et al. [10] showed that among asthmatic subjects, those with CRSwNP presented more poorly controlled asthma, increased and less reversible airway obstruction, and more marked airway inflammation than those without CRSwNP.

The clinical course of asthma in patients with CRSwNP is usually persistent and progressive and more difficult to treat than in asthmatics without CRSwNP [9, 11]. A recent study by Du and colleagues [12] has demonstrated non-asthmatic CRSwNP patients may have small airway dysfunction, which is associated with type 2 inflammation [12] and is suggestive of an asthmatic phenotype in CRSwNP patients [13]. It has been shown that asthma remains undiagnosed in 25% of patients with CRSwNP [14]; and on evaluation of bronchial hyperresponsiveness 28–40% of adults with CRSwNP were found to have previously undiagnosed asthma [15].

As the persistence of asthma is usually associated with the severity of disease, as reflected by increased bronchial responsiveness and irreversible airflow obstruction in patients with CRSwNP [11], early identification of comorbid asthma in these patients is imperative, for improved overall management of disease. As CRSwNP often precedes asthma [3], rhinologists may play an important role in screening for comorbid asthma in patients with CRSwNP. However, the factors associated with comorbid asthma in patients with CRSwNP have not been fully investigated, and a simple and practical tool for screening asthma comorbidity in this patient group is presently not available.

Thus, this study aimed to investigate factors associated with comorbid asthma in patients with CRSwNP, based on medical history and clinical examinations; and to develop a scoring system for screening asthma comorbidity in these patients.

## Methods

### Subjects and study protocol

This was a cross-sectional study, in which patients with CRSwNP aged ≥ 18 years referred to Beijing Tongren Hospital from Nov 2016 to Oct 2018 were enrolled consecutively and grouped as patients with or without asthma.

A diagnosis of CRSwNP was confirmed by a rhinologist according to the criteria of the European Position Paper on Rhinosinusitis and Nasal Polyps guidelines (EPOS) [1] and the presence of asthma was diagnosed by a respiratory physician according to the criteria of Global Initiative for Asthma (GINA) 2016.All patients with asthma were stable and recorded asthma control test (ACT) scores ≥ 20 [16]. Patients were excluded from the study if they met any of the following criteria: (1) receiving systemic steroid therapy in the proceeding 4 weeks. (2) Receiving immunosuppressive therapy or biologics treatment. (3) Diagnosed with chronic obstructive pulmonary disease, or other lung disease such as pneumonia, lung cancer, allergic bronchopulmonary aspergillosis, active pulmonary tuberculosis and interstitial lung disease. (4) diagnosed with immunodeficiency or autoimmune disease. (5) Had fungal sinusitis, inverted papilloma, or other nasal diseases that could affect results of the study. (6) had severe heart failure or significant kidney or liver dysfunction.

At the time of enrolment, demographic data and clinical history of all patients were collected using standard questionnaires. Body mass index (BMI) between 25 ~ 29.9 kg/m^2^ was defined as overweight, and ≥ 30 kg/m^2^ as obesity. Personal history of allergic diseases; including allergic rhinitis, urticaria, eczema; and non-steroidal anti-inflammatory drugs (NSAIDs) hypersensitivity were determined according to typical clinical features and/or specific tests, and the patients were examined for various clinical parameters as detailed below.

**Fig. 1:**
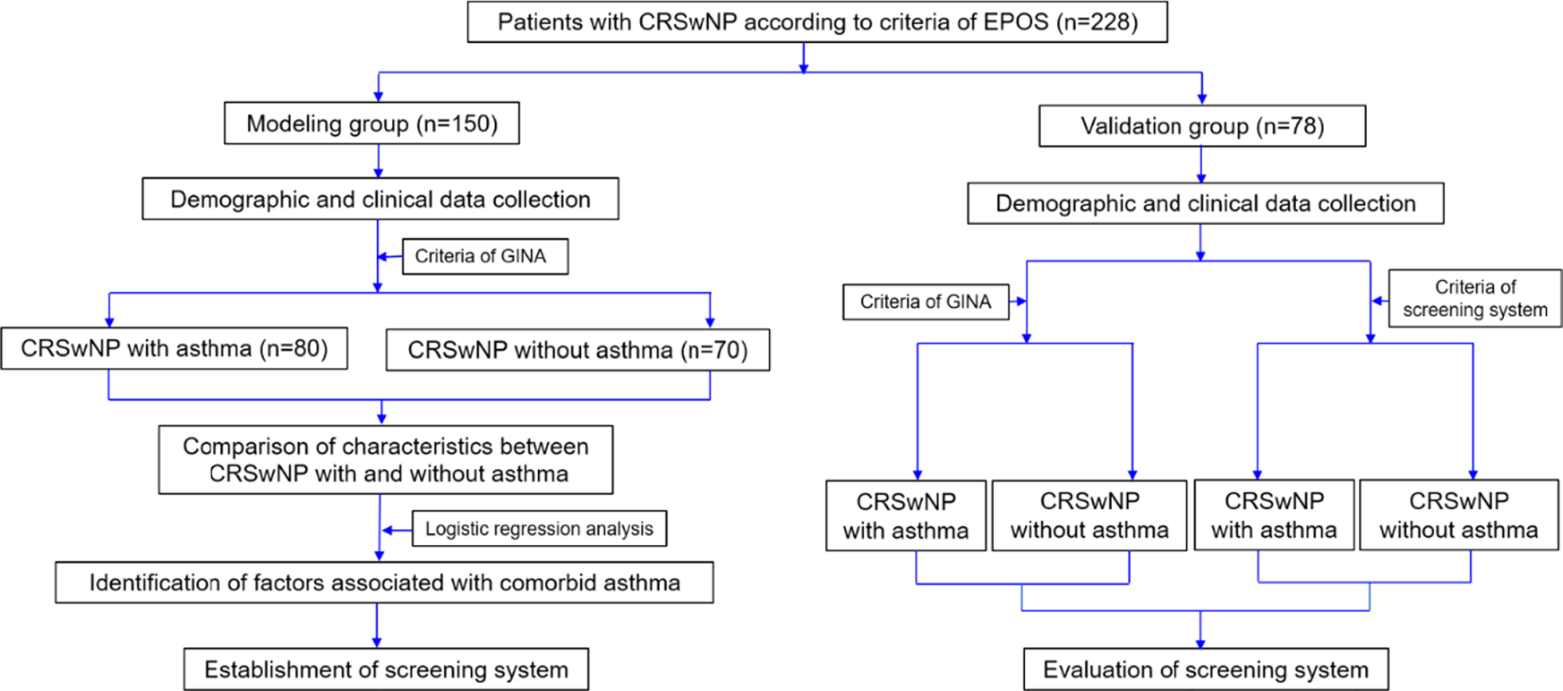
Study Flowchart. CRSwNP, chronic rhinosinusitis with nasal polyps; EPOS, European Position Paper on Rhinosinusitis and Nasal Polyps; GINA, Global Initiative for Asthma

Logistic regression analysis was used to determine the risk factors of asthma comorbidity in patients based on demographic and clinical data collected, and subsequently a scoring system was established to screen for comorbid asthma. The efficiency of the screening system was evaluated in a second cohort of CRSwNP patients enrolled from Nov 2018 to Dec 2019. The subject selection and study process are shown in Fig. 1. The study was approved by the local ethics committee of Beijing Tongren Hospital, Capital Medical University, and written informed consent was obtained from all patients prior to enrolment.

### Personal history of allergic disease

Allergic rhinitis (AR) was determined by typical clinical features (rhinorrhea, sneezing, obstruction, and pruritus) and sensitivity to at least one common aeroallergen (dust mites, pollen, cat, dog, cockroach, mold), identified by positive skin prick test (SPT) and/or serum antigen-specific immunoglobulin (Ig) E test [17]. Urticaria was determined by recurrent hives and/or angioedema, accompanied by itching, pain, or burning, which were generally relieved within 72 h [18, 19]. Eczema was diagnosed when associated with atopy. Non-steroidal anti-inflammatory drugs (NSAIDs) hypersensitivity was defined according to a history of NSAIDs-exacerbated respiratory disease (rhinitis/asthma) and/or cutaneous disease (urticaria/ angioedema) [20].

### Evaluation of eosinophils in peripheral blood and nasal secretion

Blood samples were drawn in vacutainer tubes with ethylene diamine tetra-acetic acid.

(EDTA) as anticoagulant (Ref. 368,274, Becton Dickinson, USA) and leukocyte counts and eosinophil differentiation in the anticoagulated blood samples were performed on an automatic blood analyzer (sysmex XN10B3, Japan). Nasal secretions were collected by swabbing the mucosa of middle nasal passage and inferior turbinate and applied to a slide for Wright’s staining [21]. The sample was considered to be positive when eosinophils were detected.

### Serum IgE detection and atopy determination

An automatic immunoassay system (ImmunoCap TM100, Pharmacia Company, Sweden) was used according to the manufacturer′s directions. The lower limits of detection of serum total (T)-IgE and allergen specific IgE were 2 kU/L and 0.01 kU/L, respectively. Levels of serum T-IgE > 60 kU/L and allergen specific IgE > 0.35 kU/L were considered to be increased [22]. Atopy was assessed by serum levels of specific IgE (s-IgE) to a panel of common aeroallergens (dust mites, pollen, cat, dog, cockroach, mold) and food allergens (albumen, milk, peanut, meat, seafood), and atopy was confirmed when the level of serum s-IgE to any allergen tested was > 0.35 kU/L.

### Fractional exhaled nitric oxide test

Fractional exhaled nitric oxide (FeNO) was measured using a nitric oxide analyzer (Niox; Aerocrine, Solna, Sweden) at a flow rate of 50 mL/s through the oral cavity. The gas was continuously routed into the analyzer via a side port. The procedure was repeated three times and a mean concentration of FeNO was calculated [23].

### Analysis of inflammatory cells in nasal polyp tissues

Nasal polyp tissues were dehydrated, embedded in paraffin, and then cut at 4 μm thickness by using a Leica RM2235 cryostat (Leica Microsystems, Bannockburn, Ill). The sections were stained by hematoxylin and eosin (H&E) stain and examined by using optical microscopy at a magnification of × 400. For each section, absolute numbers and percentages of eosinophils, neutrophils, plasma cells, and lymphocytes were recorded as mean of 3 non-overlapping regions, and the final evaluation for each patient’s tissue sample was recorded as mean data of 5 sections [24]. Inflammatory cell counts were performed by an experienced pathologist who was blinded to the study design. The eosinophilic characteristics of nasal polyps were evaluated according to eosinophil ≥ 10% [25], and > 27% [24].

### Pulmonary function tests

Spirometry (JAEGER, MasterScreen-body + diffusion + APS, Germany) was performed to determine the lung function measurements; including forced expiratory volume in one second (FEV_1_)/forced vital capacity (FVC)% and FEV_1_%predicted.

### Statistical analysis

For assessment of risk factor and establishment of screening system (modelling), sample size for each group was determined to be at least 10 times the number of independent risk factors. For validation of the screening system, the sample size for each group was estimated to be at least one half of the sample size needed for modelling. All statistical analyses were performed using the SPSS 23.0 statistical software package (Chicago, IL, USA). Data were expressed as mean ± standard deviation (for normal distribution parameters) or as median and 25th–75th percentiles (for abnormal distribution parameters). T test (for normally distributed parameters) and Mann–Whitney U test (for abnormally distributed parameters) were used for comparisons of continuous data between different groups. Categorical variables were analyzed by χ^2^ test. Risk factors for asthma were analyzed in modelling group with multivariate logistic regression analysis. In order to facilitate the calculation of scores, the independent risk factors of the quantitative variables obtained were converted into dichotomic variables. The smallest partial regression coefficient (β) was counted as a reference variable, and given a value of “1”. The score of other variables was obtained by dividing the partial regression coefficient by β of the reference variable [26, 27] to form a screening system for comorbid asthma. Receiver operating characteristic (ROC) curve was constructed according to the score to evaluate the screening system, and the optimal cut-off point was established by means of the Yoden Index. To further evaluate the efficiency of the screening system, asthma comorbidity was determined in the validation group and according to GINA criteria, respectively. The consistency between the two methods in diagnosing asthma was assessed according to the Kappa value; and consistency was defined as poor if Kappa ≤ 0.2, ordinary if 0.2 < Kappa ≤ 0.4, moderate if 0.4 < Kappa ≤ 0.6, good if 0.6 < Kappa ≤ 0.8, and perfect if Kappa > 0.8 [28]. *P* < 0.05 was considered to be significant.

## Results

### Demographic and clinical characteristics of the study population

A total of 228 patients with CRSwNP were enrolled as two separate cohorts, comprising the “modelling” group and the validation group (Fig. 1). The modelling group consisted of 150 patients (55 females (36.7%) and 95 males (63.3%)) with a mean age of 48 years (Table 1). Overall, 29.3% of these patients were smokers, 12.7% had a history of NSAIDs hypersensitivity, 29.3% history of allergic rhinitis, 22.7% history of urticaria, and 4% history of eczema. The average percentage and blood eosinophil counts were 5% and 0.3 × 10^9^/L, respectively; and eosinophils were found in the nasal secretions of 31.3% of the patients. The median of serum T-IgE was 103.5 kU/L, and atopy was shown in 38.7% of the patients. The level of FeNO was found to be 27 (16, 48) ppb (Table 1). Inflammatory cell classification of nasal polyps was analyzed in 126 patients with CRSwNP; including 79 patients with asthma and 47 patients without asthma. 80.2% of patients had ≥ 10% eosinophils and 63.5% of patients > 27% eosinophils. Assessment of lung function demonstrated mean FEV_1_/FVC and FEV_1_%pred to be 83.4% and 93.1%, respectively. Topical corticosteroids, leukotriene receptor antagonist, and antihistamines were used by 80%, 48%, and 40% of the patients, respectively (Table2).

**Table 1:** Baseline and clinical characteristics of patients with CRSwNP in the modelling group

Parameter	Whole group (n = 150)	CRSwNP with asthma (n = 80)	CRSwNP without asthma (n = 70)	*P* value
Female, n (%)	55 (36.7)	39 (48.8)	16 (22.9)	0.001*
Age, years	48.0 (38.0, 56.0)	48.0 (35.3, 55.0)	48.5 (41.0, 59.0)	0.239
BMI, kg/m^2^	24.2 (22.2, 27.1)	23.5 (21.6, 26.2)	25.2 (23.1, 27.7)	0.019*
Overweight (BMI = 25 ~ 29.9 kg/m^2^), n (%)	49 (32.7%)	21(26.3)	28 (40.0)	0.073
Obesity (BMI ≥ 30 kg/m^2^), n (%)	14 (9.3%)	7(8.8%)	7 (10%)	0.793
Smokers, n (%)	44 (29.3)	21 (26.3)	23 (32.9)	0.375
Pack-years smoked	15.0 (9.4, 30.0)	10.0 (7.5, 25.0)	20.0 (10.0, 40.0)	0.110
NSAIDs hypersensitivity, n (%)	19 (12.7)	17 (21.3)	2 (2.9)	0.001*
History of allergic rhinitis, n (%)	44 (29.3)	31 (38.8)	13 (18.6)	0.007*
History of urticaria, n (%)	34 (22.7)	23 (28.7)	11 (15.7)	0.057
History of eczema, n (%)	6 (4.0)	4 (5.0)	2 (2.9)	0.802
Nasal obstruction duration, years	4.0 (1.0, 10.0)	4.0 (2.0, 8.0)	3.0 (1.0, 10.0)	0.549
Blood eosinophil percentage, %	5.0 (3.1, 7.7)	6.1 (4.1, 9.2)	4.5 (2.0, 6.2)	0.001*
Blood eosinophil count, × 10^9^/L	0.3 (0.2, 0.5)	0.4 (0.3, 0.6)	0.3 (0.1, 0.4)	< 0.001*
Positive eosinophil of nasal secretion, n (%)	47 (31.3)	31 (38.8)	16 (22.9)	0.036*
Serum T-IgE, kU/L	103.5 (38.8, 244.0)	140.0 (73.0, 310.8)	54.2 (30.3, 131.0)	< 0.001*
Atopy, n (%)	58 (38.7)	33 (41.3)	25 (35.7)	0.487
Dust mite sensitivity, n (%)	43 (28.7)	27 (33.8)	16 (22.9)	0.141
Pollen sensitivity, n (%)	16 (10.7)	7 (8.8)	9 (12.9)	0.416
Animal sensitivity, n (%)	5 (3.3)	4 (5.0)	1 (1.4)	0.447
Cockroach sensitivity, n (%)	8 (5.3)	3 (3.8)	5 (7.1)	0.577
Mold sensitivity, n (%)	3 (2.0)	3 (3.8)	0 (0.0)	0.293
Food sensitivity, n (%)	21 (14.0)	11 (13.8)	10 (14.3)	0.925
FeNO, ppb	27.0 (16.0, 48.0)	35.0 (21.5, 56.8)	20.5 (13.0, 29.0)	< 0.001*

Data are presented as No. (%) for qualitative variables, as median (25th, 75th percentiles) for abnormal distribution quantitative parameters

*CRSwNP* Chronic rhinosinusitis with nasal polyps; *BMI* Body mass index; *NSAIDs* Non-steroidal anti-inflammatory drugs; *T-IgE* Total Immunoglobulin E; *FeNO* Fractional exhaled nitric oxide; *ppb* Parts per billion

* Statistical significance between CRSwNP with asthma and CRSwNP without asthma

**Table 2:** Inflammatory cell classification of nasal polyps, spirometry and medication of patients with CRSwNP for modelling

Parameter	Whole group (n = 150)	CRSwNP with asthma (n = 80)	CRSwNP without asthma (n = 70)	*P* value
*Inflammatory cell type in nasal polyps*^***†***^				
Lymphocyte, cells/HP	76.9 (40.0, 150.0)	71.0 (39.3, 122.0)	82.7 (44.0, 180.0)	0.232
Lymphocyte, %	39.3 ± 22.6	37.1 ± 21.2	42.9 ± 24.6	0.163
Plasma cel, cells/HP	30.0 (16.4, 52.8)	30.0 (18.7, 58.7)	30.0 (10.0, 40.0)	0.279
Plasma cell, %	12.5 (6.6, 20.9)	14.4 (6.8, 24.7)	10.3 (5.3, 18.2)	0.162
Eosinophil, cells/HP	80.0 (27.0, 200.0)	80.0 (30.0, 201.8)	70.0 (15.3, 180.0)	0.563
Eosinophil, %	37.3 (12.9, 65.1)	38.8 (16.7, 66.7)	30.9 (9.7, 54.5)	0.160
Neutrophil, cells/HP	0.7 (0.0, 10.0)	0.3 (0.0, 10.0)	3.7 (0.0, 20.0)	0.183
Neutrophil, %	0.3 (0.0, 3.9)	0.3 (0.0, 3.6)	1.3 (0.0, 7.1)	0.219
Eosinophil ≥ 10%, n (%)	101 (80.2)	67 (84.8)	34 (72.3)	0.090
Eosinophil > 27%, n (%)	80 (63.5)	54 (68.4)	26 (55.3)	0.142
*Spirometry*				
FEV1/FVC, %	83.4 (80.4, 89.0)	81.3 (68.3, 85.9)	82.9 (82.8, 90.9)	< 0.001*
FEV1%predicted, %	93.1 ± 17.8	87.2 ± 19.4	99.9 ± 12.8	< 0.001*
*Medication before enrolment*				
Not taking drugs, n (%)	22 (14.7)	1 (1.3)	21 (30.0)	< 0.001*
Topical corticosteroids, n (%)	120 (80.0)	79(98.8)	41 (58.6)	< 0.001*
Leukotriene receptor antagonist, n (%)	72 (48.0)	45 (56.3)	27 (38.6)	0.031*
Antihistamines, n (%)	60 (40.0)	28 (35.0)	32(45.7)	0.181

Data are presented as No. (%) for qualitative variables, as mean ± standard deviation for normal distribution quantitative parameters, as median (25th, 75th percentiles) for abnormal distribution quantitative parameters

*CRSwNP* Chronic rhinosinusitis with nasal polyps; *HP* High power; *FEV*_*1*_ Forced expiratory volume in 1 s; *FVC* Forced vital capacity

^**†**^ Totally nasal polyp tissues from 126 patients with CRSwNP were analyzed, including 79 patients with asthma and 47 patients without

^*^ statistical significance between CRSwNP with asthma and CRSwNP without asthma

Table 3 shows the demographic and clinical characteristics of CRSwNP patients enrolled as the “validation” group. Overall, this group consisted of 78 patients, of whom 32 were female (41%) and 46 male (59%), with a mean age of 48.2 years. 16.7% of these patients were smokers, and 25.6% had a history of allergic rhinitis. The mean percentage and blood eosinophil counts were 5.8% and 0.4 × 10^9^/L, respectively, and the median serum T-IgE level113 kU/L in this patient group.

**Table 3:** Baseline and clinical characteristics of patients with CRSwNP enrolled for validation of screening system

Parameter	Whole group (n = 78)	CRSwNP with asthma (n = 39)	CRSwNP without asthma (n = 39)	*P* value
Female, n (%)	32 (41.0)	23 (59.0)	9 (31.1)	0.001*
Age, years	48.2 ± 12.1	48.1 ± 13.0	48.3 ± 11.4	0.941
BMI, kg/m^2^	24.8 ± 3.1	24.0 ± 3.0	25.6 ± 3.0	0.027*
Smokers, n (%)	13 (16.7)	4 (10.3)	9(23.1)	0.129
Pack-years smoked	11.3 (10.0, 28.1)	16.3 (10.6, 21.9)	10.0 (4.8, 30.0)	0.665
History of allergic rhinitis, n (%)	20 (25.6)	13 (33.3)	7 (17.9)	0.120
Nasal obstruction duration, years	2.0 (1.0, 8.0)	3.0 (1.0, 8.0)	2.0 (1.0, 10.0)	0.984
Blood eosinophil percentage, %	5.8 ± 3.6	7.4 ± 3.9	4.2 ± 2.4	< 0.001*
Blood eosinophil count, × 10^9^/L	0.4 ± 0.2	0.5 ± 0.3	0.3 ± 0.1	< 0.001*
Serum T-IgE, kU/L	113.0 (49.8, 264.5)	191.0 (100.0, 367.0)	64.2 (34.0, 172.0)	< 0.001*

Data are presented as No. (%) for qualitative variables, as mean ± standard deviation for normal distribution quantitative parameters, as median (25th, 75th percentiles) for abnormal distribution quantitative parameters

*CRSwNP* Chronic rhinosinusitis with nasal polyps; *BMI* Body mass index; *T-IgE* Total immunoglobulin E

* Statistical significance between CRSwNP with asthma and CRSwNP without asthma

### Comparison of characteristics between CRSwNP patients with and without asthma

150 of patients with CRSwNP in the modelling group consisted of 80 patients with asthma, of whom 91.2% (73/80) had adult-onset asthma; and 70 patients without asthma. The group of patients with asthma comprised significantly more females (*P* = 0.001), had lower body mass index (BMI) (*P* = 0.019), higher prevalence of NSAIDs hypersensitivity (*P* = 0.001) and allergic rhinitis (P = 0.007), higher levels of eosinophil in blood (*P* ≤ 0.001) and nasal secretions (*P* = 0.036), serum T-IgE (*P* < 0.001), and FeNO (*P* < 0.001) compared to patients without asthma (Table 1). Spirometry parameters, including FEV_1_/FVC% and FEV_1_%pred were significantly lower in patients with asthma (both *P* < 0.001), and a significantly greater number of these patients took topical corticosteroids (*P* < 0.001) and leukotriene receptor antagonist (*P* = 0.031) than patients without asthma (Table 2). However, the two groups of patients were not significantly different with regard to age, smoking status, a history of urticaria and eczema, nasal obstruction duration, atopy, inflammatory cell classification and eosinophilic characteristics of nasal polyps (Table 1 and Table 2).

In the case of the 78 patients with CRSwNP in the validation group, there were 39 patients with asthma and 39 without. Comparison of the characteristics between CRSwNP patients with and without asthma in this group showed similar findings to those for the modelling group, apart from the finding for the history of allergic rhinitis, which was not found to be statistically significant (*P* = 0.120) (Table 3).

### Analysis of factors associated with comorbid asthma in CRSwNP patients in the modelling group

The 9 clinical variables found to be statistically significantly different between the patients with and without asthma in the modelling group (Table 1), were subjected to logistic regression analysis. This demonstrated that female gender (Odds ratio [OR] = 3.08, *P* = 0.005), history of allergic rhinitis (OR = 3.087, *P* = 0.008), levels of serum T-IgE (OR = 1.002, *P* = 0.012) and blood eosinophil count (OR = 28.845, *P* < 0.001) were found to be independent factors associated with asthma comorbidity in patients with CRSwNP (Table 4).

**Table 4:** Factors associated with comorbid asthma in patients with CRSwNP for modelling

Variables	β	OR	95%CI	*P* value
Female	1.125	3.080	1.393–6.807	0.005
History of allergic rhinitis	1.127	3.087	1.344–7.089	0.008
Serum T-IgE	0.002	1.002	1.001–1.004	0.012
Blood eosinophil count	3.362	28.845	4.932–168.713	< 0.001

*CRSwNP* Chronic rhinosinusitis with nasal polyps; *T-IgE* Total immunoglobulin E; *β* Partial regression coefficient; *OR* Odds ratio; *CI* Confidence interval

### Establishment of a scoring system to screen comorbid asthma in patients with CRSwNP

**Fig. 2:**
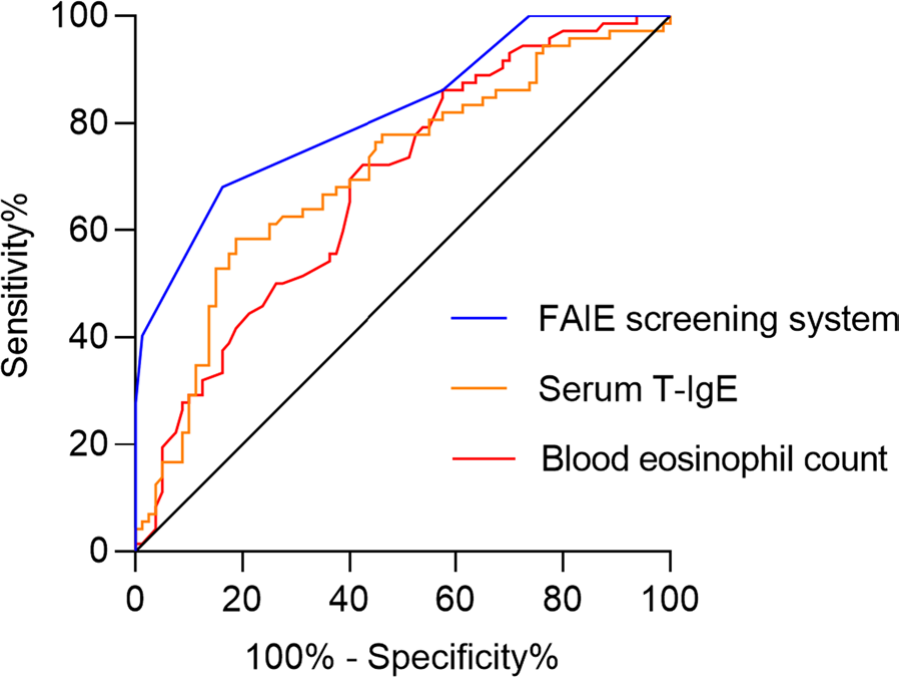
ROC curves of T-IgE, eosinophil count and FAIE screening system for assessing comorbid asthma. ROC, receiver operating characteristic; T-IgE, total immunoglobulin E; FAIE, Female gender, Allergic rhinitis, IgE, and Eosinophils as asthma risk factors

**Fig. 3:**
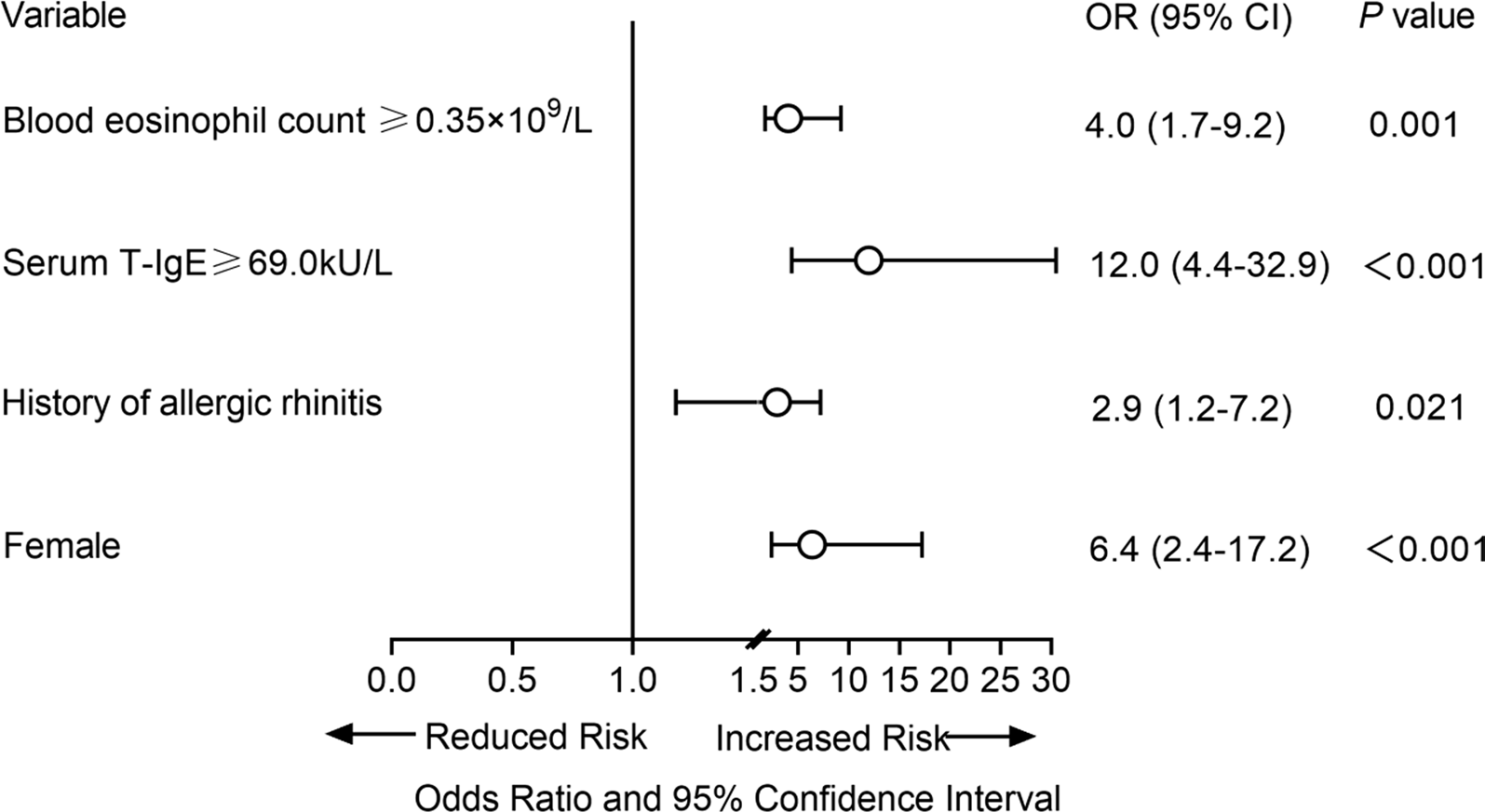
Factors associated with asthma in CRSwNP patients after conversion of quantitative variables into dichotomic variables. CRSwNP, chronic rhinosinusitis with nasal polyps; T-IgE, total immunoglobulin E; CI, confidence interval

ROC analysis demonstrated a cut-off value of 69.0 kU/L for serum T-IgE (area under curve [AUC]—ROC 0.705; sensitivity 81.3%; specificity 58.6%), and 0.35 × 10^9^/L of blood eosinophil count (AUC-ROC 0.685; sensitivity 57.5%; specificity 72.9%) were optimal for determination of comorbid asthma in patients with CRSwNP (Fig. 2). Following conversion of these quantitative variables into dichotomic ones, four variables, including female gender, history of allergic rhinitis, serum T-IgE ≥ 69.0 kU/L, and blood eosinophil count ≥ 0.35 × 10^9^/L were found to be independent factors associated with comorbid asthma (β 1.852, OR 6.4, *P* < 0.001; β 1.070, OR 2.9, *P* = 0.021; β 2.483, OR 12.0, *P* < 0.001; β 1.388, OR 4.0, *P* = 0.001, respectively) (Fig. 3). Each variable was given a different scoring point according to its β value (Table 5), and a scoring system (FAIE; Female, Allergic rhinitis, IgE, and Eosinophil) ranging from 0 to 6; with 0 = no risk and 6 = high risk; was established accordingly to screen for comorbid asthma in patients with CRSwNP.

**Table 5:** Screening system (FAIE) for comorbid asthma in patients with CRSwNP

Factors	Points
*Female*	
Yes	2
No	0
*History of allergic rhinitis*	
Yes	1
No	0
Serum T-IgE	
≥ 69.0 kU/L	2
< 69.0 kU/L	0
*Blood eosinophil count*	
≥ 0.35 × 10^9^/L	1
< 0.35 × 10^9^/L	0

*CRSwNP* Chronic rhinosinusitis with nasal polyps; *FAIE* Female, Allergic rhinitis, IgE, and Eosinophils as asthma risk factors; *T-IgE* Total immunoglobulin E

### Efficiency of FAIE system for screening comorbid asthma in patients with CRSwNP

The area under ROC for the FAIE system was found to be 0.823 (Fig. 2). The sensitivity, specificity, positive and negative predictive values, and prevalence of asthma at different cut-off points of the system are shown in Table 6. Based on the Youden Index, the optimal cut-off point of the system was ≥ 3; with sensitivity of 83.8% and specificity of 68.6%. Employing this system, 75.3% of the patients with score ≥ 3 were identified as patients with asthma according to GINA criteria.

**Table 6:** Screening efficiency of FAIE system for comorbid asthma in CRSwNP patients in the modelling group

FAIE score	≥ 0	≥ 1	≥ 2	≥ 3	≥ 4	≥ 5	≥ 6
Sensitivity (%)	100.0	100.0	98.8	83.8	42.5	26.3	5.0
Specificity (%)	0.0	28.6	41.4	68.6	87.1	100.0	100.0
Positive predictive value (%)	53.3	61.5	65.8	75.3	79.1	100.0	100.0
Negative predictive value (%)	0.0	100.0	96.7	78.7	57.0	54.3	47.9
Youden Index	0.0	0.286	0.402	0.524	0.296	0.263	0.050
Prevalence of asthma (%)	53.3	61.5	65.8	75.3	79.1	100.0	100.0

*CRSwNP* Chronic rhinosinusitis with nasal polyps; *FAIE* Female, Allergic rhinitis, IgE, and Eosinophils as asthma risk factors

Similar results were also found for the patients in the validation group. Based on the Youden Index, the optimal cut-off point of the system was also ≥ 3; with sensitivity and specificity of 76.9% and 74.4%, respectively. Furthermore, 75% of the patients with score ≥ 3 were identified as patients with asthma according to GINA criteria (Table 7). The asthma comorbidity determined with FAIE score ≥ 3 points in the validation group, was also comparable and moderately consistent with that defined by GINA criteria (Kappa = 0.513, *P* < 0.001) (Table 8).

**Table 7:** Screening efficiency of FAIE system for comorbid asthma in CRSwNP patients in the validation group

FAIE score	≥ 0	≥ 1	≥ 2	≥ 3	≥ 4	≥ 5	≥ 6
Sensitivity (%)	100.0	100.0	98.8	76.9	48.7	35.9	20.5
Specificity (%)	0.0	27.8	40.3	74.4	94.9	94.9	97.4
Positive predictive value (%)	52.6	60.6	64.8	75.0	90.5	87.5	88.9
Negative predictive value (%)	0.0	100.0	96.7	76.3	64.9	59.7	55.1
Youden Index	0.0	0.278	0.391	0.513	0.436	0.308	0.179
Prevalence of asthma (%)	52.6	60.6	64.8	75.0	90.5	87.5	88.9

*CRSwNP* Chronic rhinosinusitis with nasal polyps; *FAIE* Female gender, Allergic rhinitis, IgE, and Eosinophils as asthma risk factors

**Table 8:** Consistency of comorbid asthma determined by FAIE system and GINA criteria in patients for validation

FAIE system criteria	GINA criteria		Total
	CRSwNP with asthma	CRSwNP without asthma	
CRSwNP with asthma	30	10	40
CRSwNP without asthma	9	29	38
Total	39	39	78

*FAIE* Female gender, Allergic rhinitis, IgE, and Eosinophils as asthma risk factors; *GINA* Global initiative for asthma; *CRSwNP* Chronic rhinosinusitis with nasal polyps

## Discussion

In this cross-sectional study, risk factors associated with comorbid asthma in CRSwNP patients were explored based on medical history and clinical examinations. The study demonstrated that female gender, history of allergic rhinitis, increased levels of serum T-IgE (≥ 69.0 kU/L) and blood eosinophil count (≥ 0.35 × 10^9^/L) were independent risk factors for comorbid asthma in patients with CRSwNP. A scoring system based on these risk factors was also established for screening comorbid asthma, and preliminarily findings suggest that this system may have satisfactory screening efficiency. To the best of our knowledge, this is the first study to systematically evaluate the factors associated with comorbid asthma in patients with CRSwNP, and to establish a system to screen comorbid asthma in these patients.

Previous studies on risk factors of asthma incidence have shown differences between childhood-onset and adult-onset asthma [29–31]. As asthma is a heterogeneous disease with different underlying mechanisms, exploring risk factors associated with a certain phenotype may be more helpful for precise identification and management of individuals at high-risk. Asthmatics with comorbid CRSwNP are characterized by more severe and persistent asthma, worse quality of life and tend to show fixed airflow limitation compared with asthmatics without CRSwNP [15]. Furthermore, asthma is more difficult to control and more exacerbation prone in the presence of nasal polyposis. Indeed, nasal polyposis has been shown to be a predictor of chronicity and persistence of new-onset asthma [11, 32]. As nasal disease often precedes asthma [11], it is important to identify and treat asthma early in patients with CRSwNP in order to maintain the lung function within normal range, and decrease risk of future asthma exacerbation in these patients. However, the onset of asthma in CRSwNP patients seems to be cryptogenic, as the patients may be asymptomatic and a large proportion of patients are undiagnosed [12, 13, 15]. Indeed, even rhinologists may sometimes mistake the symptoms of cough and dyspnea as resulting from CRSwNP rather than from asthma. Consequently, comorbid asthma in patients with CRSwNP may be easily neglected, especially at early stage [14, 15, 33]. Therefore, it is necessary to explore the risk factors associated with comorbid asthma in patients with CRSwNP, and establish a practical system for especially rhinologists to screen the patients at high-risk.

Previous studies have shown that a female gender, allergic rhinitis, increased serum T-IgE, and blood eosinophil count are associated with increased asthma incidence, and that co-existence of these factors may further increase the risk of asthma. Many epidemiological studies suggest that women are at increased risk of developing adult-onset asthma and also suffer from more severe disease than men [30, 34, 35]. One early meta-analysis demonstrated that females with nasal polyps were more likely to have asthma than males [36]. The current study has also demonstrated that, female gender is one of several independent risk factors for comorbid asthma in patients with CRSwNP, with 91.2% of the patients having adult-onset asthma.

Our findings for blood eosinophil counts and serum T-IgE levels are also in accordance with the findings of Staikūniene and colleagues [8], who found that patients with CRSwNP and comorbid asthma had significantly higher blood eosinophil counts and serum T-IgE levels than patients with only CRSwNP. A systemic review and meta–analysis by Bao and colleagues [37] has shown that serum IgE level ≥ 60 kU/L is a risk factor for developing asthma in either preschool children or those at early school age. Blood eosinophil count has also been shown to be associated with asthma incidence. Backman and colleagues [38] have demonstrated that in infancy, a low blood eosinophil count < 0.25 × 10^9^/L on admission for bronchiolitis was a significant protective factor, and a high blood eosinophil count > 0.45 × 10^9^/L on convalescence was a significant risk factor for asthma in adulthood independently from atopic status [38]. Similarly, Bai and colleagues [39] have demonstrated that a high blood eosinophil count > 110 cells/μL is a risk factor for incident asthma [39]. In the current study, blood eosinophilia has also been shown to be an independent risk factor for comorbid asthma in patients with CRSwNP. As both serum IgE and blood eosinophil count were shown to be independent risk factors for comorbid asthma in CRSwNP patients, this suggests that this disease may possibly be due to enhanced activation and interaction of two branching pathways of type 2 inflammation; i.e. Interleukin (IL)-5/eosinophilic and IL-4/IL-13/IgE. In this context, it would be interesting to explore whether decreasing levels of eosinophil and IgE, or inhibiting their activities (for example, with biologics) might delay or attenuate the incidence of asthma in patients with CRSwNP.

Both allergic and nonallergic rhinitis have been shown to be independent risk factors for adult-onset asthma [40]. Interestingly, Guerra and colleagues [40] showed that the association between rhinitis and asthma appeared to be stronger among female than among male patients. Moreover, the association between rhinitis and asthma appeared to be stronger in subjects with high total IgE levels than in patients with intermediate and low IgE levels [40]. These findings also support the findings from the present study, which demonstrated as female, allergic rhinitis, and increased serum IgE to be independent risk factors for comorbid asthma in CRSwNP patients.

Many CRSwNP patients with comorbid asthma have been shown to be characterized by type 2 airway inflammation [15, 41]. Recent evidence indicates that increased FeNO may be associated with increased risk of exacerbation and poor asthma control [42, 43]. In the current study, CRSwNP patients with comorbid asthma showed higher levels of FeNO compared to patients without asthma; however, FeNO was not identified as an independent risk factor for asthma. It could be due to several reasons. First, it has been demonstrated that about 30% of patients with CRSwNP show elevated FeNO comparable with asthmatics, but cannot be diagnosed as asthma [13], and thus may minimize the difference in FeNO between CRSwNP patients with and without asthma. Second, in the current study the levels of FeNO may be decreased by more applications of inhaled corticosteroids in patients with CRSwNP and comorbid asthma [44]. As the level of FeNO has been shown to be modestly associated with level of blood eosinophils [45], blood eosinophil count as an independent risk factor may to some degree also represent the role of FeNO in predicting the risk of comorbid asthma in CRSwNP patients.

The FAIE system developed in the current study was shown to have better screening efficiency for comorbid asthma when the cut-off value of ≥ 3 points was used, both in modelling and validation groups, with sensitivity and specificity of approximately 80% and 70% respectively, and 75% of patients with definite asthma were included. However, the sensitivity of the system to screen comorbid asthma was shown to increase to 98.8% when the cut-off value of ≥ 2 points was used, suggesting that FAIE score ≥ 2 points may be taken as a predictor of comorbid asthma in patients with CRSwNP. Moreover, for CRSwNP patients with FAIE score ≥ 2 points, careful inquiry of asthma symptoms and use of lung function tests may be helpful in identifying patients with comorbid asthma earlier; whereas for CRSwNP patients with FAIE score ≥ 3 points, consultation with a respiratory specialist may be needed based on clinical manifestations and examination of the patients.

The findings from this study are somewhat limited**.** The predicted probability of asthma for a given patient, using a logistic regression model rather than just a 0–6 score, would probably have been more accurate if predictors such as T-IgE and blood EOS were included as continuous measures rather than dichotomous measures. For example, in the score calculation, a T-IgE of 68 would give 0 points but a T-IgE of 70 would give 2 points, and thus some information may be lost with this simplification. Nevertheless, there is a case to be made for the advantages of a simplified scoring system that can be easily calculated from information readily obtainable in clinic. The relatively small sample size might also lead to some data deviation and bias of case enrolment, particularly as this was a cross-sectional single-centre study. Furthermore, some factors such as sputum inflammatory cells, access to health care, family history of asthma or atopy, and menopausal status in women, which may be associated with comorbid asthma, were not included. The finding that despite greater topical corticosteroids and leukotriene receptor antagonist use by patients with CRSwNP and comorbid asthma, the levels of eosinophils and IgE were found to be significantly higher in these patients than in CRSwNP patients without asthma, suggests that the influence of greater medication use also needs to be taken into consideration and these findings need to be confirmed in prospective multicentre studies with large sample size in the future.

## Conclusions

Female gender, history of allergic rhinitis, increased levels of serum T-IgE and blood eosinophil count are important risk factors for development of comorbid asthma in patients with CRSwNP. The FAIE system appears to be a useful and practical screening system for asthma comorbidity, and should be helpful for early diagnosis and prompt management of asthma in patients with CRSwNP. To observe the development of asthma in CRSwNP patients who have not yet been diagnosed with comorbid asthma should be especially helpful in evaluating the efficiency of the system for predicting asthma incidence in CRSwNP patients.

## Data Availability

The complete dataset is included in this manuscript.

## Abbreviations

ACT: Asthma control test
AR: Allergic rhinitis
AUC: Area under curve
BMI: Body mass index
CI: Confidence interval
CRSwNP: Chronic rhinosinusitis with nasal polyps
CT: Computed tomography
EDTA: Ethylene diamine tetra-acetic acid
EPOS: European position paper on rhinosinusitis and nasal polyps
FeNO: Fractional exhaled nitric oxide
FEV_1_: Forced expiratory volume in one second
FVC: Forced vital capacity
GINA: Global initiative for asthma
NSAIDs: Non-steroidal anti-inflammatory drugs
OR: Odds ratio
ROC: Receive operating characteristics
SPT: Skin prick test
s-IgE: Specific immunoglobulin E
T-IgE: Total immunoglobulin E
